# Multiple Administration of Endogenous Amines TIQ and 1MeTIQ Protects Against a 6-OHDA-Induced Essential Fall of Dopamine Release in the Rat Striatum: In Vivo Microdialysis Study

**DOI:** 10.1007/s12640-017-9824-8

**Published:** 2017-10-26

**Authors:** Agnieszka Wąsik, Irena Romańska, Agnieszka Zelek-Molik, Lucyna Antkiewicz-Michaluk

**Affiliations:** 1 Department of Neurochemistry, Institute of Pharmacology, Polish Academy of Sciences, Smętna 12, 31-343 Kraków, Poland; 2 Department of Brain Biochemistry, Institute of Pharmacology, Polish Academy of Sciences, Smętna 12, 31-343 Kraków, Poland

**Keywords:** Tetrahydroisoquinolines (TIQs), 6-Hydroxydopamine (6-OHDA), Microdialysis study, Parkinson’s disease (PD)

## Abstract

Parkinson’s disease (PD) represents one of the neurodegenerative disorders which are caused by degeneration of dopaminergic neurons in the nigrostriatal pathway. Different toxins, e.g., 6-hydroxydopamine (6-OHDA), are used to model PD in animals. 6-OHDA is a neurotoxin which damages catecholaminergic neurons via production of oxygen radicals. Tetrahydroisoquinolines (TIQs) are endogenous amines which are present in the mammalian brain. Some of them, like TIQ and 1-methyl-1,2,3,4-tetrahydroisoquinoline (1MeTIQ), demonstrate neuroprotective properties. These compounds act as reversible MAO inhibitors and this way block free radical formation. To continue our previous experiments, we evaluated the effect of acute and chronic treatment with TIQ and 1MeTIQ on locomotor/exploratory activity and the release of dopamine as well as its metabolite 3-methoxytyramine (3-MT) in the striatum of unilaterally 6-OHDA-lesioned and sham-operated rats using in vivo microdialysis methodology. Additionally, the changes in the concentration of tyrosine hydroxylase in the substantia nigra were measured. A unilateral 6-OHDA lesion in the substantia nigra produces a strong reduction in the release of dopamine (approx. 70%) and 3-MT (approx. 50%) in the rat striatum. This effect was completely inhibited by multiple administration of TIQ and 1MeTIQ. The results obtained from the in vivo microdialysis study suggest that multiple treatment with both endogenous amines, TIQ and 1MeTIQ, protects dopaminergic neurons against a 6-OHDA-induced deficit of dopamine release. Furthermore, these amines were able to maintain physiological functions of striatal dopamine neurons damaged by a unilateral 6-OHDA lesion.

## Introduction

Parkinson’s disease (PD) is a neurodegenerative disorder which affects approximately 6 million people in the world. It is caused by degeneration of dopaminergic neurons in the nigrostriatal pathway which results in a strong reduction in dopamine concentration in the striatum (Hornykiewicz 1989; Schapira et al. 1990). Parkinsonian patients develop characteristic motor symptoms such as rigidity, bradykinesia, resting tremor, and postural instability (Ossowska 1994). There are a lot of data suggesting various mechanisms of neuronal degeneration in PD, e.g., oxidative stress, mitochondrial dysfunction, formation of free radicals, neuroinflammation and glial activation, excitotoxicity, and genetic and environmental factors. (Dykens 1999; Jenner 2003; Schapira and Jenner 2011).

Tetrahydroisoquinolines (TIQs) belong to a group of endogenous amines which are present in the mammalian brain (Antkiewicz-Michaluk et al. 2001). Some of them, such as salsolinol or 1BnTIQ, show neurotoxic activity, while others (especially 1-methyl-1,2,3,4-tetrahydroisoquinoline—1MeTIQ) demonstrate neuroprotective properties. Our earlier studies have shown that both TIQ and 1MeTIQ inhibited activity of two types of monamine oxidases (MAO-A and MAO-B) and increased monoamine neurotransmitter levels in the brain (Patsenka and Antkiewicz-Michaluk 2004). 1MeTIQ showed neuroprotective activity both in in vitro (Antkiewicz-Michaluk et al. 2006) and in vivo studies in several animal models of Parkinson’s disease (PD) (Antkiewicz-Michaluk et al. 2003, 2004, 2014; Wąsik et al. 2016a, b). As it was demonstrated by our team, both TIQ and 1MeTIQ strongly influence dopamine metabolism without changing the rate of total dopamine catabolism but by strongly inhibiting the MAO-dependent catabolic pathway and markedly activating the catechol-*O*-methyltransferase (COMT)-dependent O-methylation (Antkiewicz-Michaluk et al. 2001; Patsenka et al. 2004). Such molecular mechanism of action leads to inhibition of free radicals production which takes place during dopamine MAO-dependent oxidation. Additionally, 1MeTIQ acts as a scavenger of free radicals (Antkiewicz-Michaluk et al. 2006). Moreover, some data indicated that the concentration of 1MeTIQ was significantly reduced in the brains of patients suffering from Parkinson’s disease and in the brains of rodents exposed to agents evoking experimental parkinsonism (Tasaki et al. 1991).

6-Hydroxydopamine (6-OHDA) is a neurotoxin which damages catecholaminergic neurons. This compound is the most widely used for modeling PD in in vivo and in vitro studies. 6-OHDA uses the same catecholamine transport system as dopamine and accumulates in the cytosol inducing neurotoxicity (Luthman et al. 1989; Gonzalez-Hernandez et al. 2004). Because 6-OHDA does not cross the blood-brain barrier, obtaining a toxic effect in the central nervous system is possible after a direct injection of this compound into the corresponding brain structures by means of stereotaxic surgery. Neurotoxic action of 6-OHDA consists of two-step mechanism: accumulation of the toxin in catecholaminergic neurons and disruption of cellular homeostasis which leads to neuronal damage (Luthman et al. 1989). Inside the neurons, 6-OHDA produces cytotoxic species through both enzymatic and non-enzymatic mechanisms. Endo-cellular trace elements, such as iron and manganese, are implicated in these processes (Cadet and Brannock 1998). Moreover, oxidation of 6-OHDA induced by MAO-A generates H_2_O_2_, what leads to the production of oxygen radicals (Cohen 1984).

Our current research is a continuation of previous experiments involving the 6-OHDA model. The aim of the present study was to evaluate the effect of acute and chronic treatment with two endogenous amines, TIQ and 1MeTIQ, on locomotor/exploratory activity and the release of dopamine and its metabolite 3-methoxytyramine (3-MT) in the striatum of unilaterally sham-operated and 6-OHDA-lesioned rats using in vivo microdialysis methodology. Additionally, the changes in the concentration of tyrosine hydroxylase in the substantia nigra were measured.

## Materials and Methods

### Animals and Treatments

All experiments were carried out on male Wistar rats with an initial body weight of 280–300 g. All animals had free access to standard laboratory food and tap water and were kept at room temperature (22 °C) under an artificial light/dark cycle (12/12 h, light on at 7:00). The rats used for the microdialysis study after surgery were housed for a couple of days individually. The experiments were carried out between 09:00 and 16:00 h. Control rats were treated with an appropriate solvent (0.9% NaCl). The rats were administered TIQ or 1MeTIQ at a dose of 50 mg/kg intraperitoneally (i.p.) either acutely (14 days after a 6-OHDA lesion) or as multiple doses (during 14 days; first injection 24 h after a 6-OHDA lesion).

All experimental procedures were carried out in accordance with the Guide for the Care and Use of Laboratory Animals issued by the National Institutes of Health and received an approval from of the Bioethics Commission as being compliant with the Polish law. All experimental procedures were approved by the Local Bioethics Commission of the Institute of Pharmacology, Polish Academy of Sciences in Kraków.

### Surgical Procedure

The rats were anesthetized with ketamine (75 mg/kg) and xylazine (10 mg/kg) and secured in a stereotaxic frame (Stoelting, USA). A stainless steel needle was inserted unilaterally through a small hole in the skull and the needle tip was placed in the right substantia nigra (SN) with the following stereotaxic coordinates: A/P − 5.5, L/M − 2.0, and H − 7.8 mm from the bregma and dura, respectively. The solution of 6-hydroxydopamine (6-OHDA) was freshly prepared immediately prior the surgery. 6-OHDA hydrochloride, at a dose of 8 μg (calculated as the free base) dissolved in a volume of 4 μl of sterile 0.9% NaCl supplemented with 0.05% ascorbic acid, was slowly infused into the right substantia nigra at a flow rate of 0.5 μl/min using a 10-μl Hamilton syringe. After termination of the 6-OHDA infusion, the cannula was left in place for further 4 min for complete diffusion of the toxin and then was slowly retracted. Sham-operated rats were treated in the same manner, but received equivalent volumes of vehicle instead of 6-OHDA.

### Drugs

6-OHDA and 1,2,3,4-tetrahydroisoquinoline (TIQ) (Sigma-Aldrich, USA) were obtained commercially. 1-Methyl-1,2,3,4-tetrahydroisoquinoline (1MeTIQ) was synthesized (according to Cannon and Webster 1958) at the Department of Drug Chemistry of the Institute of Pharmacology, the Polish Academy of Sciences in Kraków. Purity of the compounds was verified by measuring the melting point and assessing homogeneity in a chromatographic column. The compounds were dissolved in a 0.9% NaCl solution.

### Behavioral Study

#### Locomotor Activity

The locomotor activity and rearing were measured using actometers (Opto-Varimex activity monitors; Columbus Inst., USA) linked on line to an IBM-PC compatible computer. Each cage (43 × 44 × 25 cm) was surrounded by a 15 × 15 array of photocell beams located 3 cm from the floor surface, as reported previously (Filip et al. 2007). Interruptions of these photocell beams were counted as a measure of horizontal locomotor activity defined as the distance traveled (in cm). Horizontal locomotor activity was recorded for 45 min (exploratory activity) and analyzed using the Auto-Track Software Program (Columbus Inst., USA). TIQ (50 mg/kg i.p.) or 1MeTIQ (50 mg/kg i.p.) was administered acutely or chronically. The control group was treated with saline. Immediately after TIQ or 1MeTIQ injections, the animals were transferred to the experimental cages. Seven animals from each group were subjected to behavioral analysis.

### Biochemical Study

#### In Vivo Microdialysis

Rats were anesthetized with ketamine (75 mg/kg) and xylazine (10 mg/kg) and secured in a stereotaxic frame (Stoelting, USA). Vertical microdialysis guide cannulas (Intracerebral Guide Cannula with Stylet; BAS Bioanalytical, USA) were implanted into the right striatum (STR) according to the following stereotaxic coordinates: A/P + 1.0, L/M − 2.5, and V/D − 3.5 mm from the bregma and dura (G. Paxinos and C.H. Watson). Implantation of the cannula was performed immediately after 6-OHDA lesions during the same operation/anesthesia. Fourteen days after the surgery, microdialysis probes were inserted into the cannulas and the striatum was perfused with an artificial cerebrospinal fluid (aCSF) consisting of 140 mM NaCl, 2.7 mM KCl, 1.2 mM CaCl_2,_ 1 mM MgCl_2,_ 0.3 mM NaH_2_PO_4_, and 1.7 mM Na_2_HPO_4_ (pH 7.4) at a flow rate of 1.5 μl/min with a microinfusion pump (Stoelting, IL, USA). Samples were collected from freely moving rats at 20-min intervals after a 3-h wash-out period. Control and lesioned (6-OHDA) animals received saline injections and dialysis samples were collected for 180 min. In the mixed groups, 1MeTIQ or TIQ (50 mg/kg i.p.) was administered acutely (the first administration 14 days after a 6-OHDA lesion) or chronically (for 14 consecutive days; the first administration 24 h after a 6-OHDA lesion). All dialysates were immediately frozen on dry ice (− 70 °C) until use in a biochemical assay.

Levels of dopamine (DA) and its extraneuronal metabolite 3-MT were assayed in dialysates (20 μl) using HPLC (Coulochem III, Germany) with electrochemical detection, as described below. Chromatographic data were processed using the Chromeleon Dionex computer program (Germany). Dopamine and its metabolites were quantified by chromatograph peak height in comparison with standard run on the day of analysis. At the end of the experiment, frozen brains were examined histologically for correct probe placement. Each group consisted of six animals.

#### Immunoblotting

Protein was extracted through high-speed shaking in plastic tubes with stainless steel beads in a tissue lyser with 100 μl of ice-cold RIPA lysis buffer (Sigma, USA), containing a complete mini protease inhibitor (Roche Diagnostics, USA). After incubation for 30 min, the homogenates were centrifuged at 10,000*g* for 20 min at 4 °C. The resulting supernatants were collected and subjected to protein analysis using the Bicinchoninic Acid Assay Kit (Sigma, USA). Equal amounts of protein extracts (12 μg) were boiled in Laemmli buffer containing 5% β-mercaptoethanol for 5 min, separated through SDS-PAGE (4–15%) and transferred to nitrocellulose membranes. The membranes were blocked with 5% nonfat dry milk in Tris-buffered saline containing Tween-20 (TBST; pH = 7.6) for 1 h at room temperature and incubated overnight at 4 °C with a primary antibody against tyrosine hydroxylase (1:2000; Cell Signaling, USA) or α-synuclein (1:2000; Cell Signaling, USA). After three washes with the blocking solution, the membranes were incubated with the appropriate secondary antibodies for 1 h at room temperature, followed by three washes with TBST. Antibody binding was detected using an enhanced chemiluminescence kit (ECL Plus, Pierce, USA). Equal loading of protein per sample was further confirmed after probing with anti-calnexin antiserum (CNX; 1:5000; Enzo Life Sciences, USA) or anti-β-actin antiserum (1:5000; Sigma, USA). All Western blot analyses were performed at least twice to confirm the results. The chemiluminescence signal was visualized using a luminescent image analyzer Fuji-Las 4000 (Fuji, Japan). Immunoreactive bands were quantified using an image analyzer (ScienceLab, MultiGauge V3.0).

## Calculations and Statistics

The results from the locomotor activity and immunoblotting were analyzed by a one-way analysis of variance (ANOVA) followed by Duncan’s post hoc test, when appropriate. The data from microdialysis studies were analyzed by a one-way analysis of variance (ANOVA) for repeated measures, followed by (if significant differences arose) Duncan’s post hoc test.

## Results

### Behavioral Tests

#### Locomotor Activity

**Fig. 1:**
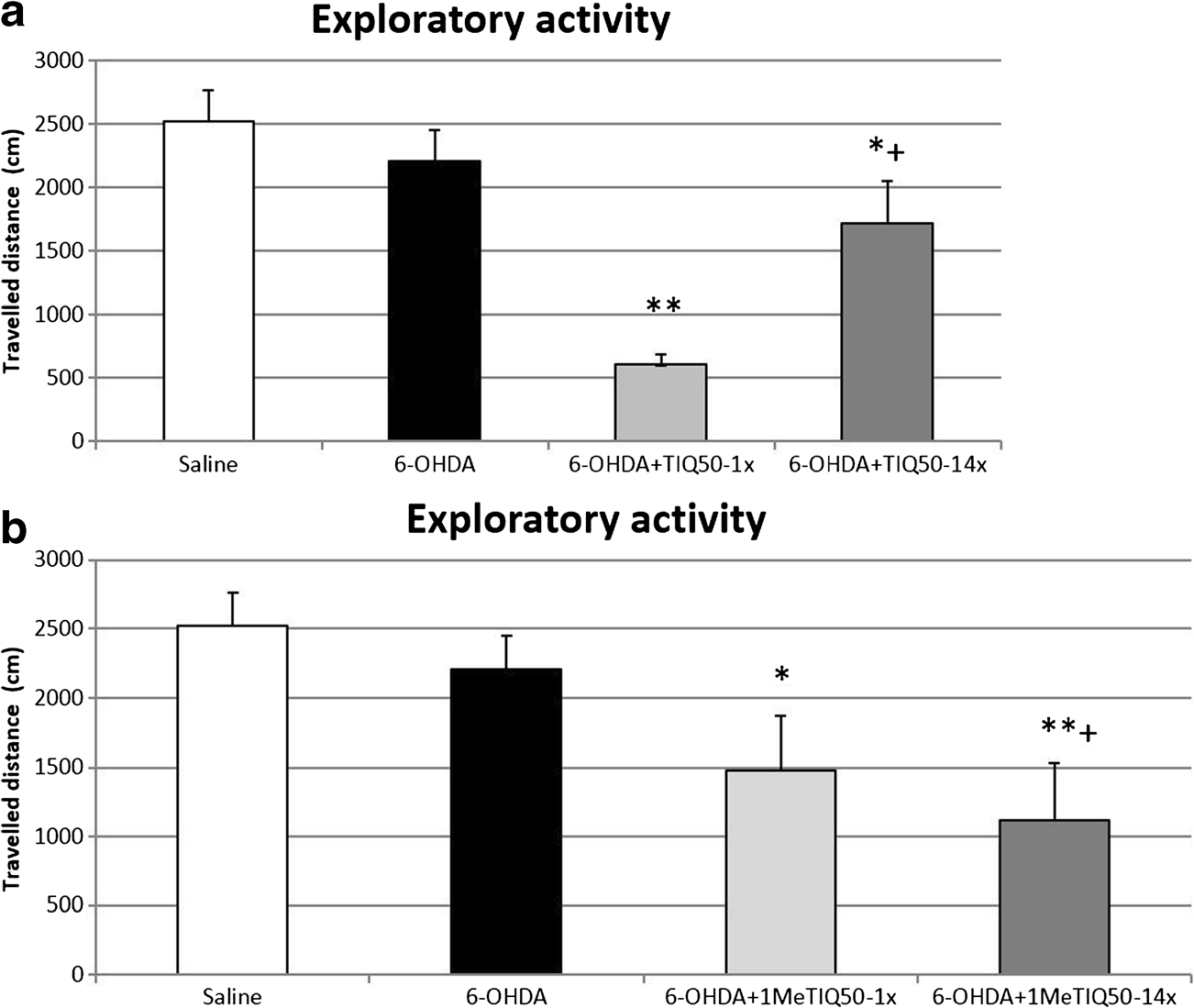
The effect of acute or chronic administration of TIQ (**a**) or 1MeTIQ (**b**) on 6-OHDA-induced changes in exploratory activity of rats. Sham-operated rats received a single injection of saline (control). 6-OHDA was infused unilaterally into the right substantia nigra (8 μg) 14 days before measurement of exploratory activity. In the mixed group, TIQ (**a**) or 1MeTIQ (**b**) (50 mg/kg i.p.) was administered acutely (on the day of the experiment) or chronically for 14 consecutive days. Rats were placed into actometers immediately after the treatment. Movements were recorded for 45 min. The data are expressed as means ± SEM (*n* = 6 animals). The data were analyzed with a one-way ANOVA, followed by Duncan’s post hoc test. Statistical significance *p < 0.05, **p < 0.01 vs. saline-treated group; ^+^ *p <* 0.05, vs. 6-OHDA-lesioned group

A one-way ANOVA showed that locomotor activity of rats was slightly decreased in the 6-OHDA-lesioned group, but the result did not reach statistical significance (Fig. 1a, b). In addition, in the joint treatment group, when TIQ was given acutely (50 mg/kg) combined with a 6-OHDA lesion, we observed a significant decrease (*p* < 0.01) in the exploratory activity of rats during the first 45 min of the experiment (Fig. 1a). Similar but weaker effect was observed in the lesioned group after chronic TIQ (50 mg/kg) administration (*p* < 0.05). Reduced locomotor activity was also observed in the mixed group with a 6-OHDA lesion when 1MeTIQ (50 mg/kg) was given both acutely and chronically. However, the strongest effect was observed after chronic administration of 1MeTIQ combined with a 6-OHDA lesion (*p* < 0.01) (Fig. 1b).

### Biochemical Study

#### Dopamine Concentration in the Extracellular Space*:* In Vivo Microdialysis Study

##### The Effect of a Unilateral 6-OHDA Lesion

**Fig. 2:**
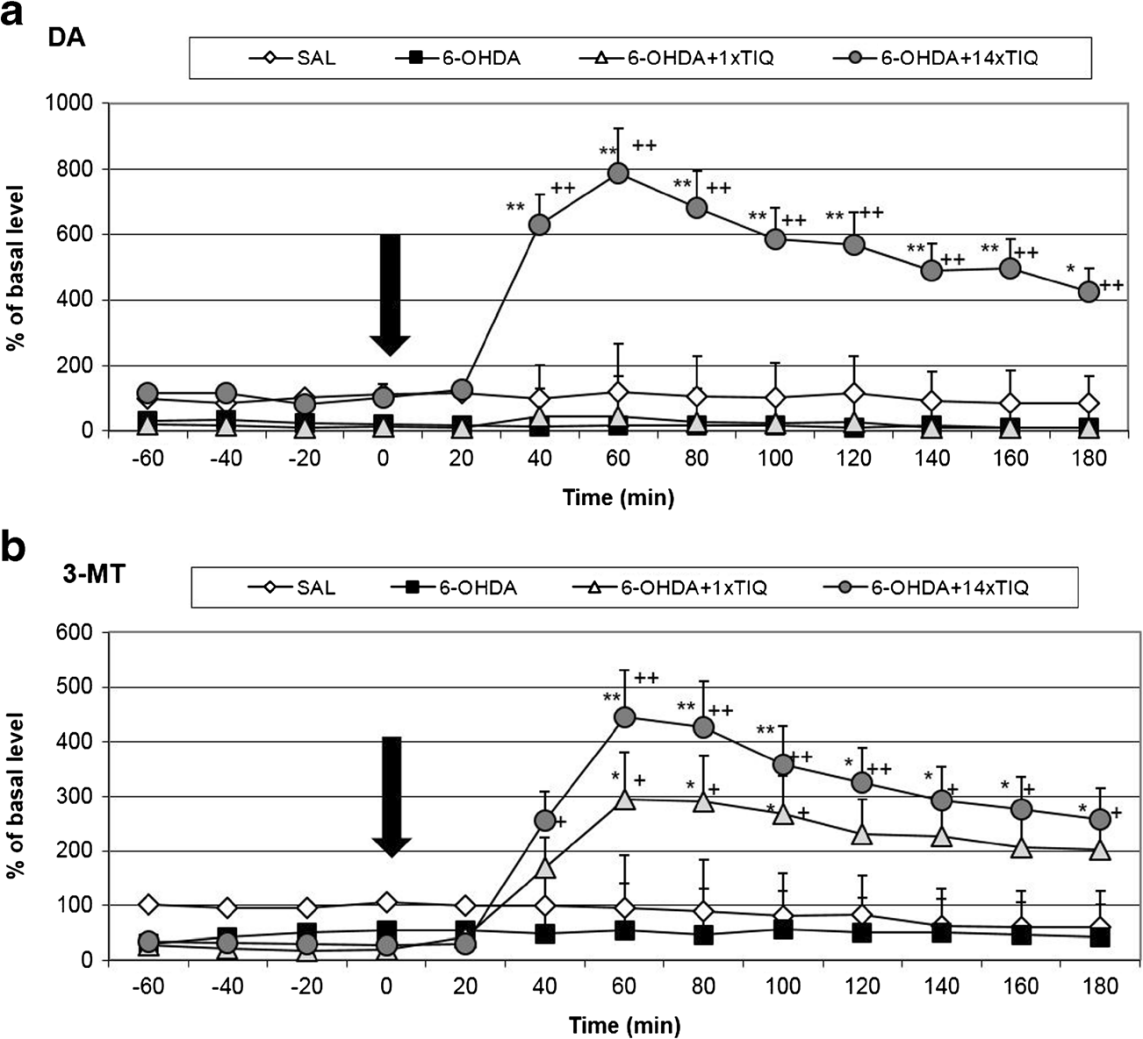
The effect of acute or chronic administration of TIQ on 6-OHDA-induced changes in the dopamine release (**a**) and 3-MT concentration (**b**) in the rat striatum. Sham-operated rats received a single injection of saline (control). 6-OHDA was infused unilaterally into the right substantia nigra (8 μg) 14 days before microdialysis study. In the mixed group, TIQ (50 mg/kg i.p.) was administered acutely (on the day of the experiment) or chronically for 14 consecutive days. The dialysate was collected every 20 min. The concentration of dopamine (DA) (**a**) or 3-MT (**b**) was measured. The data are expressed as means ± SEM (*n* = 5–6). Statistical significance *p < 0.05, ^++^ *p <* 0.01 **p < 0.01 from the basal value (Duncan’s test); ^+^ *p* < 0.05, ^++^ *p* < 0.01 vs. the 6-OHDA-lessioned group

**Fig. 3:**
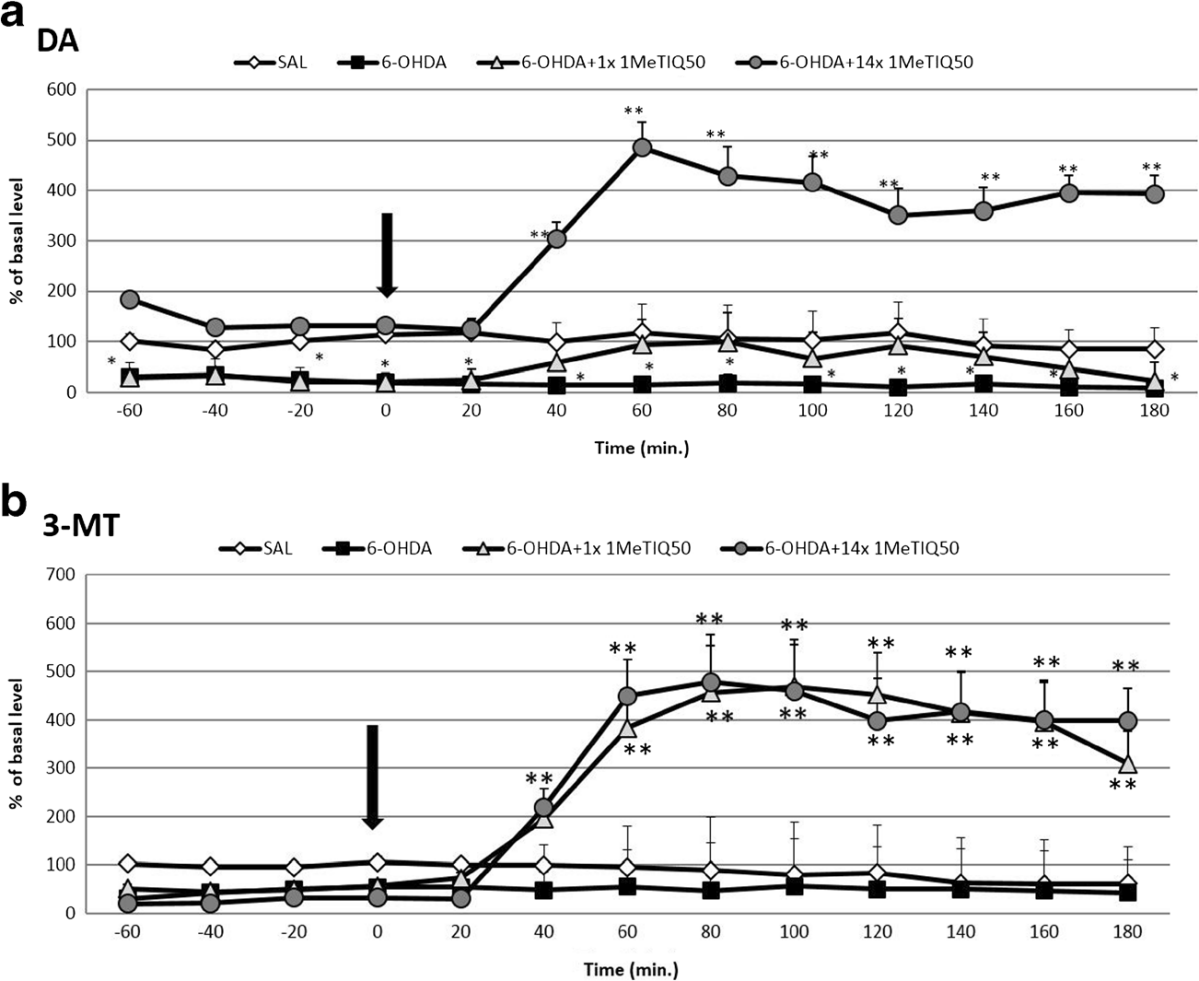
The effect of acute or chronic administration of 1MeTIQ on 6-OHDA-induced changes in the dopamine release (**a**) and 3-MT concentration (**b**) in the rat striatum. Sham-operated rats received a single injection of saline (control). 6-OHDA was infused unilaterally into the right substantia nigra (8 μg) 14 days before microdialysis study. In the mixed group, 1MeTIQ (50 mg/kg i.p.) was administered acutely (on the day of the experiment) or chronically for 14 consecutive days. The dialysate was collected every 20 min. The concentration of dopamine (DA) (**a**) or 3-MT (**b**) was measured. The data are expressed as means ± SEM (*n* = 5–6). Statistical significance *p < 0.05, **p < 0.01 from the basal value (Duncan’s test)

The mean control basal extracellular concentration of dopamine in dialysates obtained from the striatum was approximately 7.9 ± 2.1 (pg/20 μl). A statistical analysis demonstrated a significant reduction in the extracellular dopamine concentration (about 70% fall, *F*[3,16] = 18.21; *p* < 0.01) in the basal samples (from − 60 to 0 min) 14 days after a unilateral 6-OHDA lesion in comparison with the control group (saline) (Figs. 2a and 3a). The effect was observed until the end of the experiment.

##### The Effect of Single or Chronic Administration of TIQ on 6-OHDA-Induced Changes in the Dopamine Release in the Rat Striatum

A one-way ANOVA for repeated measures indicated a significant effect of treatment with TIQ (*F*[3,17] = 8.83; *p* < 0.01) (Fig. 2a). A post hoc analysis demonstrated that an acute injection of TIQ (50 mg/kg i.p.) made 14 days after 6-OHDA did not change the toxic effect of the lesion and the concentration of dopamine decreased about 70% in comparison to that of the control group (Fig. 2a). In contrast, chronic administration of TIQ (50 mg/kg i.p.) completely prevented the toxic effect of 6-OHDA and restored the dopamine concentration to the control level. Moreover, after the last injection of TIQ, the dopamine release was strongly increased—up to 800% (Fig. 2a).

##### The Effect of Single or Chronic Administration of 1MeTIQ on 6-OHDA-Induced Changes in the Dopamine Release in the Rat Striatum

The analysis of variance indicated a significant effect of treatment with 1MeTIQ (*F*[3,16] = 18.21; *p* < 0.01). Duncan’s test demonstrated that an acute dose of 1MeTIQ (50 mg/kg i.p.) given 14 days after 6-OHDA inhibited the toxic effect of the lesion and returned the dopamine concentration up to the control level (from 60 to 160 min) (Fig. 3a). In addition, in the combined treatment group, repeated injections of 1MeTIQ (50 mg/kg i.p.) completely blocked the effect induced by a 6-OHDA lesion (Fig. 3a). The concentration of dopamine in basal samples was slightly above the control (saline) group, and after the final injection of 1MeTIQ, the dopamine release increased by over 400% (Fig. 3a).

#### 3-MT Concentration in the Extracellular Space

##### The Effect of a Unilateral 6-OHDA Lesion

The statistical analysis indicated that a unilateral 6-OHDA-induced lesion of the substantia nigra produced a significant effect on 3-MT concentration in the rat striatum (*F*[3,15] = 4.81; *p* < 0.05) (Figs. 2b and 3b). Duncan’s test showed that a 6-OHDA lesion reduced the level of 3-MT by approximately 50% in comparison to the control (saline) group throughout the entire measurement period (Figs. 2b and 3b).

##### The Effect of Single or Chronic Administration of TIQ on 6-OHDA-Induced Changes in the Concentration of 3-MT in the Rat Striatum

A one-way ANOVA for repeated measures revealed a significant effect of treatment with TIQ (*F*[3,15] = 5.62; *p* < 0.05) (Fig. 2b) on the concentration of 3-MT in the rat striatum. Acute TIQ (50 mg/kg i.p.) administration produced a significant (*p* < 0.05) increase in the concentration of 3-MT in the rat striatum (up to 300%). A post hoc analysis indicated that chronic administration of TIQ produced stronger effect and the concentration of 3-MT was elevated up to 450% (*p* < 0.01) (Fig. 2b).

##### The Effect of Single or Chronic Administration of 1MeTIQ on 6-OHDA-Induced Changes in the Concentration of 3-MT in the Rat Striatum

The analysis of variance indicated a significant effect of treatment with 1MeTIQ (*F*[3,15] = 4.81; *p* < 0.05) in the 6-OHDA-lesioned rats. Duncan’s test demonstrated that both acute and chronic administration of 1MeTIQ (50 mg/kg i.p.) produced a strong (about 500%) and significant (*p* < 0.01) increase in the striatal concentration of 3-MT in the 6-OHDA-lesioned groups (Fig. 3b).

#### Immunoblotting

**Fig. 4:**
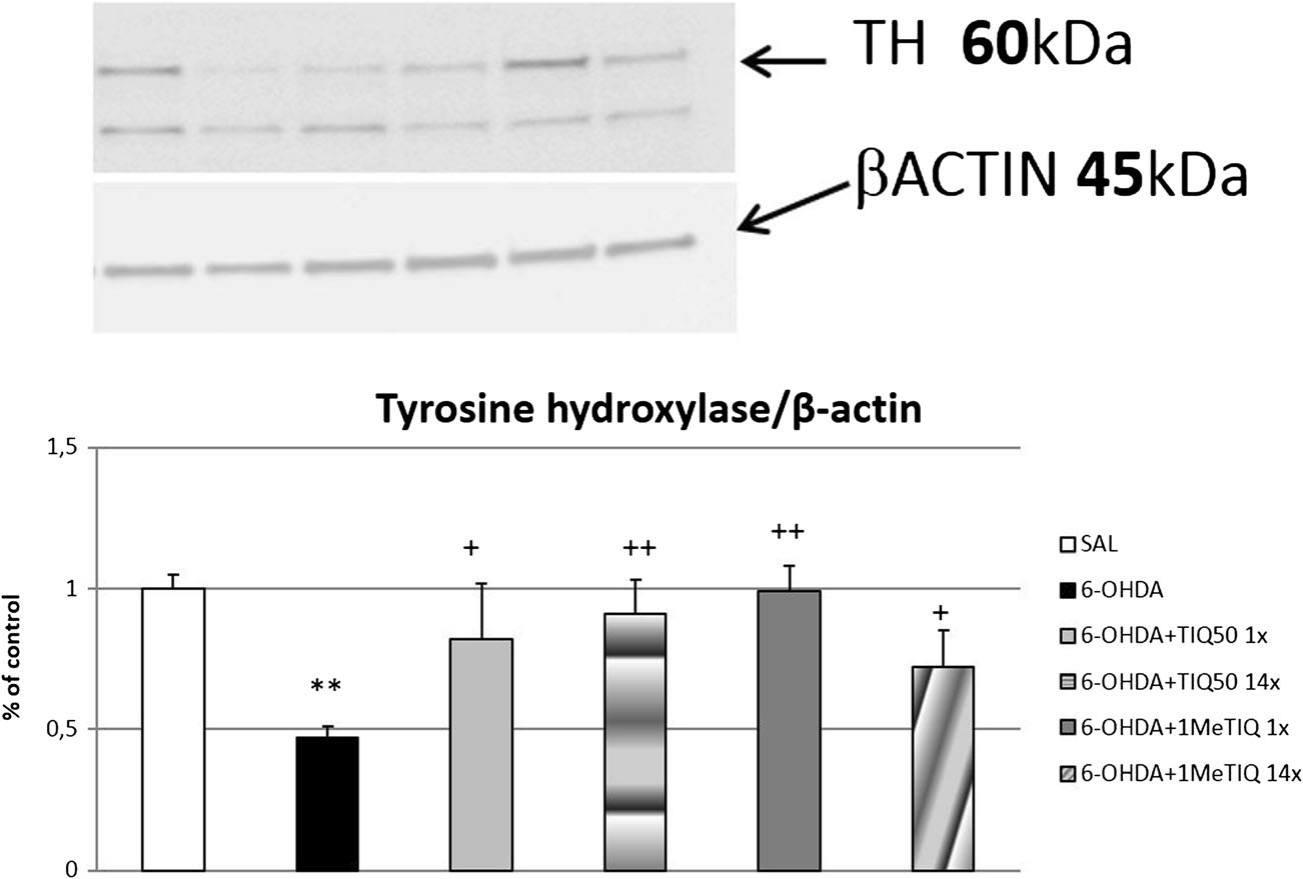
The effect of acute or chronic administration of TIQ or 1MeTIQ on 6-OHDA-induced changes in the concentration of tyrosine hydroxylase (TH) in the substantia nigra of the rat. Sham-operated rats received a single injection of saline (control). 6-OHDA was infused unilaterally into the right substantia nigra (8 μg) 14 days before experiment. In the mixed groups, TIQ or 1MeTIQ (50 mg/kg i.p.) was administered acutely (on the day of the experiment) or chronically for 14 consecutive days. The right substantia nigra was dissected 3 h after last TIQs administration. The data are expressed as means ± SEM (*n* = 6 animals). The data were analyzed with a one-way ANOVA, followed by Duncan’s post hoc test. Statistical significance **p < 0.01 vs. saline-treated group; ^+^ *p <* 0.05, ^++^ *p <* 0.01 vs. 6-OHDA-lesioned group

The data obtained from the ipsilateral side show that a 6-OHDA lesion significantly reduced (approx. 50%, *p* < 0.01) the level of tyrosine hydroxylase in the substantia nigra (Fig. 4). In addition, acute and chronic treatment with both TIQ (50 mg/kg) and 1MeTIQ (mg/kg) significantly inhibits the toxic effect induced by a 6-OHDA lesion (Fig. 4).

## Discussion

It is well documented that 6-OHDA acts as a neurotoxin and selectively destroys catecholaminergic neurons leading to its death (Kostrzewa et al. 2006; Szkilnik et al. 2014; Zhang et al. 2017). The data acquired in our recent in vivo microdialysis studies indicated that a unilateral 6-OHDA lesion in the substantia nigra produces strong disturbances and a reduction in the release of dopamine (about 70% fall in comparison with the control group) (Figs. 2a and 3a). The result obtained from our previous ex vivo experiment demonstrated a strong ipsilateral depletion of dopamine level in the tissues (striatum, substantia nigra) and impaired dopamine transmission following unilateral administration of 6-OHDA into the substantia nigra (Wąsik et al. 2016b).

The main finding of the present study is that multiple administration of endogenous amines, TIQ and 1MeTIQ, completely prevented disturbances in dopamine release induced by a unilateral 6-OHDA lesion. As it was demonstrated on Figs. 2a and 3a, the concentration of dopamine in the basal samples was similar to that of the control (sham + saline) group. In contrast to acute administration, both TIQ and 1MeTIQ given chronically protected dopamine neurons against 6-OHDA-generated damage. It should be noted that injections of both investigated compounds were started 24 h after 6-OHDA administration and continued up to the 14th day. In addition, multiple administration of these compounds produced a strong and long-lasting elevation of dopamine release after the last dose (about 500 to 800%) (Figs. 2a and 3a). Our earlier studies have established that both compounds exert influence on the dopamine catabolism and act as reversible MAO inhibitors blocking the MAO-dependent oxidative pathway in dopamine metabolism (Antkiewicz-Michaluk et al. 2001). Such mechanism of action not only reduces the production of free radicals in the cell but also causes an elevation of the concentration of dopamine in the synaptic cleft. As it was demonstrated in our previous investigations, both acute and chronic treatment with TIQ induced a significant increase of dopamine release (300 and 500%, respectively) in the rat striatum. In the same time, acute and chronic treatment with 1MeTIQ produced weaker effect; the elevation of dopamine release was about 200% (Wąsik et al. 2016a). 6-OHDA well penetrates into dopamine neurons using a dopamine transporter (DAT). TIQs also manifest affinity for monoaminergic transporters (Patsenka et al. 2004). We suggest that neurotoxins 6-OHDA and TIQs compete for access to DAT, so early administration of TIQs produces better therapeutic effects. Comparing the neuroprotective effect of TIQs after single and multiple administration, we clearly demonstrated that early application (24 h after 6-OHDA) of investigated compounds inhibits 6-OHDA-induced neurotoxicity and what is more, restores proper function of the dopaminergic neurons. In contrast, single administration of 1MeTIQ only slightly increased the concentration of dopamine in the extracellular space in 6-OHDA-treated rats, while acute TIQ administration did not give any protection (Figs. 2a and 3a). As it was shown by Sachs and Jonson (1975), generation of reactive oxygen species as a result of 6-OHDA autoxidation is the main molecular mechanism underlying 6-OHDA neurotoxicity. Therefore, the main neuroprotective mechanism of TIQs seems to be, as it was previously demonstrated, concerned with their ability to scavenge free radicals and inhibit their production (Antkiewicz-Michaluk et al. 2006).

Microdialysis study involving 6-OHDA-treated rats demonstrated a significant fall (approx. 50% compared to control group) in the concentration of 3-MT, an extraneuronal metabolite of dopamine (Figs. 2b and 3b). These results are consistent with other findings, as it is well known that 6-OHDA acts as an irreversible COMT inhibitor (the main enzyme responsible for conversion of DA into 3-MT) (Borchardt et al. 1976). 3-MT is considered to be the most reliable indicator of dopamine release into the synaptic cleft (Egan et al. 1991; Karoum et al. 1994). Chronic treatment with both TIQ and 1MeTIQ did not change these disturbances in the basal samples in 6-OHDA-treated rats, however, directly after acute or chronic administration, both compounds produced a significant elevation of 3-MT concentration (about 400–500%, *p* < 0.01) (Figs. 2b and 3b). As our lab has previously demonstrated, chronic treatment with both TIQ and 1MeTIQ induced a huge increase in 3-MT level (3500 and 2000%, respectively) (Wąsik et al. 2016a). It seems that effect of TIQs is connected with their ability to potentiate COMT activity (Antkiewicz-Michaluk et al. 2006). The second possible mechanism may involve inducing adaptive changes by 6-OHDA lesions, which could lead to COMT hypersensitivity. As a result, we observed strong 3-MT elevation several hours after TIQs administration. 3-MT is an active metabolite of dopamine and is capable of triggering signaling events (Antkiewicz-Michaluk et al. 2008; Alachkar et al. 2010). As it was demonstrated previously, 3-MT shows affinity for α1-adrenergic and D1 and D2 receptors as an antagonist, so it may play an important role as an inhibitory regulator counteracting excessive stimulation of catecholaminergic neurons (Antkiewicz-Michaluk et al. 2008; Alachkar et al. 2010). In the present behavioral study, we observed a decrease of the exploratory activity in 6-OHDA-treated rats 14 days after the lesion. This effect was significantly augmented by acute and multiple administration of both compounds (Fig. 1a, b). The results obtained from the locomotor activity test confirmed that high concentration of 3-MT observed after TIQs administration may act as an endogenous neuroleptic (Antkiewicz-Michaluk et al. 2008). Such mechanism of action of 3-MT may explain paradoxical effect of both TIQ and 1MeTIQ, where despite the elevation in dopamine release after the administration, the exploratory activity of rats was reduced (Fig. 1a, b). A similar effect was observed when TIQs were given into the naïve rats (Wąsik et al. 2012, 2016a).

Tyrosine hydroxylase (TH) is a key enzyme in dopamine synthesis which catalyzes transformation of l-tyrosine into l-DOPA, a precursor of dopamine, noradrenaline, and adrenaline (Molinoff and Axelrod 1971). TH is mainly expressed in dopaminergic and noradrenergic neurons in the brain (Tenkin et al. 2014; Pickel et al. 1975). Motor impairment represents one of the characteristic symptoms in animal models of Parkinson’s disease, which is a consequence of a reduction in TH in the nigrostriatal pathway (Fernandes et al. 2012; Leão et al. 2015). The data obtained in the present paper clearly indicate that a unilateral 6-OHDA lesion induced a significant decrease in the TH level on the ipsilateral side of the substantia nigra (Fig. 4). Such toxic effect of 6-OHDA was antagonized by acute and chronic treatment with both investigated compounds: TIQ and 1MeTIQ (Fig. 4). Neuroprotective potential of 1MeTIQ on TH-positive cells was also demonstrated by Parrado et al. (2000), who established in vivo the effect of 1MeTIQ on dopaminergic neuronal death induced by MPP+ by using quantitative immunochemistry to measure TH levels. These data confirm neuroprotective activity of 1MeTIQ, as it prevented neurotoxin-induced loss of TH-positive cells in the substantia nigra and striatum.

## Conclusions

The data presented in this paper and obtained from the in vivo microdialysis study suggest that multiple treatment with both endogenous amines TIQ and 1MeTIQ protects dopaminergic neurons against 6-OHDA-induced disturbances of dopamine release. Furthermore, these amines were able to maintain physiological functions of the striatal dopamine neurons damaged by a 6-OHDA lesion.
